# Tertiary lymphoid structure related B-cell IgE isotype switching and secondary lymphoid organ linked IgE production in mouse allergy model

**DOI:** 10.1186/s12865-020-00376-7

**Published:** 2020-08-07

**Authors:** Dmitrii Borisovich Chudakov, Dmitrii Yuryevich Ryasantsev, Daria Sergeevna Tsaregorotseva, Olga Dmitrievna Kotsareva, Gulnar Vaisovna Fattakhova, Elena Viktorovna Svirshchevskaya

**Affiliations:** 1 Shemyakin-Ovchinnikov Institute of Bioorganic Chemistry of RAS, 117997 16/10 Miklukho-Maklaya Street, Moscow, Russia; 2 Sechenov First Moscow State Medical University, 127994 4 Rachmanov Alley, Moscow, Russia

**Keywords:** IgE-mediated allergy, Tertiary lymphoid structures, Low allergen doses, Anti-proliferative drugs, Local IgE class switching

## Abstract

**Background:**

Numerous data obtained by different research laboratories indicate that specific IgE production is triggered independently of specific IgG or IgA ones and so it is not linked to fully matured germinal centers formation in the secondary lymphoid organs. The aim of this study was to clarify whether specific IgE production is triggered by low antigen doses administrated in tertiary tissues enriched by lymphoid structures.

**Methods:**

Ovalbumin (OVA) in different doses (100 ng to 10 μg) was administrated three times a week for 4–5 weeks intraperitoneally (i.p.) or subcutaneously (s.c.) to female BALB/c mice in the wither region which is enriched in fat-associated lymphoid clusters or in the foot pad region not containing them.

**Results:**

OVA-specific IgE was predominantly induced by low but not high antigen doses and only after immunization into the withers. IgE isotype switching was triggered exclusively in the withers adipose tissue but not in the regional lymph nodes while mature IgE expressing cells were observed both in the withers and lymph nodes. Anti-proliferative genotoxic stress inducing drugs shifted the balance from IgG1 towards IgE production.

**Conclusions:**

Tertiary lymphoid structures possess unique environment where B-cell antibody isotype switching to IgE predominantly occurs. This phenomenon is partially explained by hampered proliferation of B-cells in these structures.

## Background

There is currently a significant increase in allergic diseases around the world, especially in the developed countries [1]. These diseases include atopic dermatitis, asthma, allergic rhinitis and food allergy [1]. Arising more often in the childhood, they tend to progress over time manifesting in more severe forms in the adulthood, the phenomenon known as the atopic march [2]. The mechanism of allergy is most often associated with the production of IgE to harmless antigens [1]. The only method for its etiotropic, rather than symptomatic, therapy is allergen-specific immunotherapy (ASIT). This method is associated with the stimulation of the production of blocking allergen-specific IgG4 antibodies [3].

In order to develop new methods of ASIT dedicated to stimulate allergen-induced B-cell switching to IgG4 but not IgE isotype, it is clearly necessary to understand specific mechanisms triggering selective B-cell immunoglobulin class switching to these isotypes. For the development of the general strategy to eliminate IgE-producing B-cells and their precursors, it is important to know the exact site where B-cell IgE isotype switching occurs. We have previously shown that in young (1–8 years old) patients with allergy to house dust mite specific IgE production is not associated with specific IgG, IgG4 or IgA1 production [4] and may occur outside the germinal centers of secondary lymphoid organs (SLOs). Possibly it can occur in the tertiary lymphoid structures (TLSs) or so-called tissue-associated lymphoid clusters. Because of the close proximity to epithelial or endothelial barrier tissues, immune processes in TLSs may be somewhat different from those in SLOs. For example, type 2 innate lymphoid cells (ILC2) activated by tissue damage-induced cytokines interleukins (IL) 25, 33 or thymic stromal lymphopoetin (TSLP) [5], instead of T helper-2 cells, may act as the main producers of pro-allergic cytokines like IL-5, 13 [5] and, to a lesser extent, IL-4 [6], in TLSs during the early phases of immune response.

Lifestyle can significantly affect the severity of atopic diseases. One of the characteristics of the developed country population is the high prevalence of obesity [7]. Epidemiological data indicate a direct relationship between the degree of obesity and the severity of asthma. The molecular and cellular mechanisms of this association can be diverse. For example, in obese individuals, the production of pro-inflammatory IL-6 in tissues may be increased. Adipokine effects are also suggested. Very often, although not always, the level of leptin in patients with obesity is increased, and the level of adiponectin is reduced [7, 8]. It was shown that the administration of leptin to BALB/c mice increased the intensity of allergic inflammation and production of IgE while the administration of adiponectin inhibited it [8–10]. It is especially important to note that adipose tissue contains TLSs, although their number varies depending on the anatomical location [11]. The role of TLSs in the humoral immune response to allergens has not been studied.

Despite a large number of successively developed mouse allergy models, most of them require high allergen doses [12–17] sometimes with adjuvants like Alum [12–15] or CFA [16], resulting not only in high IgE levels but also high IgG1 [12–15] and sometimes IgG2a [17] production. So, in these models, humoral immune response differs from that in allergic patients. Among allergic individuals some of them have significant IgG4 production [4, 18, 19] which is not, however, associated with specific IgE levels [4, 18, 19]. Elevated IgG4 levels detected in atopic patients can represent secondary reaction in response to multiple antigens entering the mucosal surfaces. The presence of already existing specific IgE makes it possible to induce IgG4 production due to the immune recognition of IgE-antigen complexes [20–22].. Indeed, recent data reviewed in [23, 24] show that high antigen doses induce robust germinal center response in SLOs which could, in certain circumstances, block the development of IgE producing B-cells. It is not surprising that B-cell IgE class switch is usually detected in the SLO-like lymph nodes and specifically within germinal centers but this process is transient, since it occurs mainly at early stages of its development and not in mature structures [25, 26]. Moreover, numerous studies [27–29] provide the evidence that in allergic patients B-cell IgE class switching usually occurs in TLSs such as nasal polyps or inducible bronchial-associated lymphoid clusters [28, 30].

The aim of this work was to clarify the hypothesis that B-cell IgE class switching in response to low, but not high, administrated antigen doses occurs mostly in the specific milieu of TLSs but not in SLOs. In clinical practice allergens usually pass through the respiratory barrier tissues where inducible bronchial-associated lymphoid tissue or nasal polyps represent TLSs [28, 30]. In humans these clusters are induced during early infancy, especially among children due to their higher susceptibility to viral and bacterial infection, or to the exposure to certain substance such as diesel-derived particles [31]. These structures can be generated in young mice by the administration of specific substances such as lipopolysaccharide [32]. To avoid the difficulties of small antigen doses delivery to mouse lungs as well as to induce respiratory tract linked TLSs formation in adult animals, we chose to use a principally novel model in which antigen was administrated s.c. in the withers (s.c.w.). It is well known that the withers of mice contains significant volume of white adipose tissue and many so called fat-associated lymphoid clusters (FALCs) [33] which resemble those associated with the respiratory epithelium. Importantly, tissue damage-induced cytokines, such as IL-33 and TSLP, could be produced not only in barrier epithelium cells [5] but also in adipose tissue, particularly, in adipocytes and adipose-linked endothelium [34, 35]. Of note, at first ILC2 were discovered in the association with FALCs [36], though later ILC2 were identified in SLOs as well [5]. In adipose tissues, ILC2 not only promote allergic type 2 inflammation but also function as metabolic and homeostatic regulators [37]. In addition, the introduction of antigen into the subcutaneous adipose tissue allows one to check the role of the fat associated TLSs in the synthesis of pro-allergic antibodies. The results can be relevant to understanding the mechanisms linking the obesity and atopic march.

We demonstrate here that direct IgE class switch in response to subcutaneously administrated allergen occurs in local TLSs, and adipose tissue provides unique environment for B cell activation.

## Results

### Low dose antigen administration in TLS-enriched, but not TLS-depleted, zone induces specific IgE production

To reconstitute typical clinical situation in which allergens enter mucosal tissue over a long period, mice were repeatedly immunized s.c. with low (10 or 100 ng), intermediate (1 μg) or high (10 μg) antigen doses in the regions which are enriched in TLSs such as withers (s.c.w.) and peritoneal cavity (i.p) or lack them (foot pad, f.p.).

**Fig. 1:**
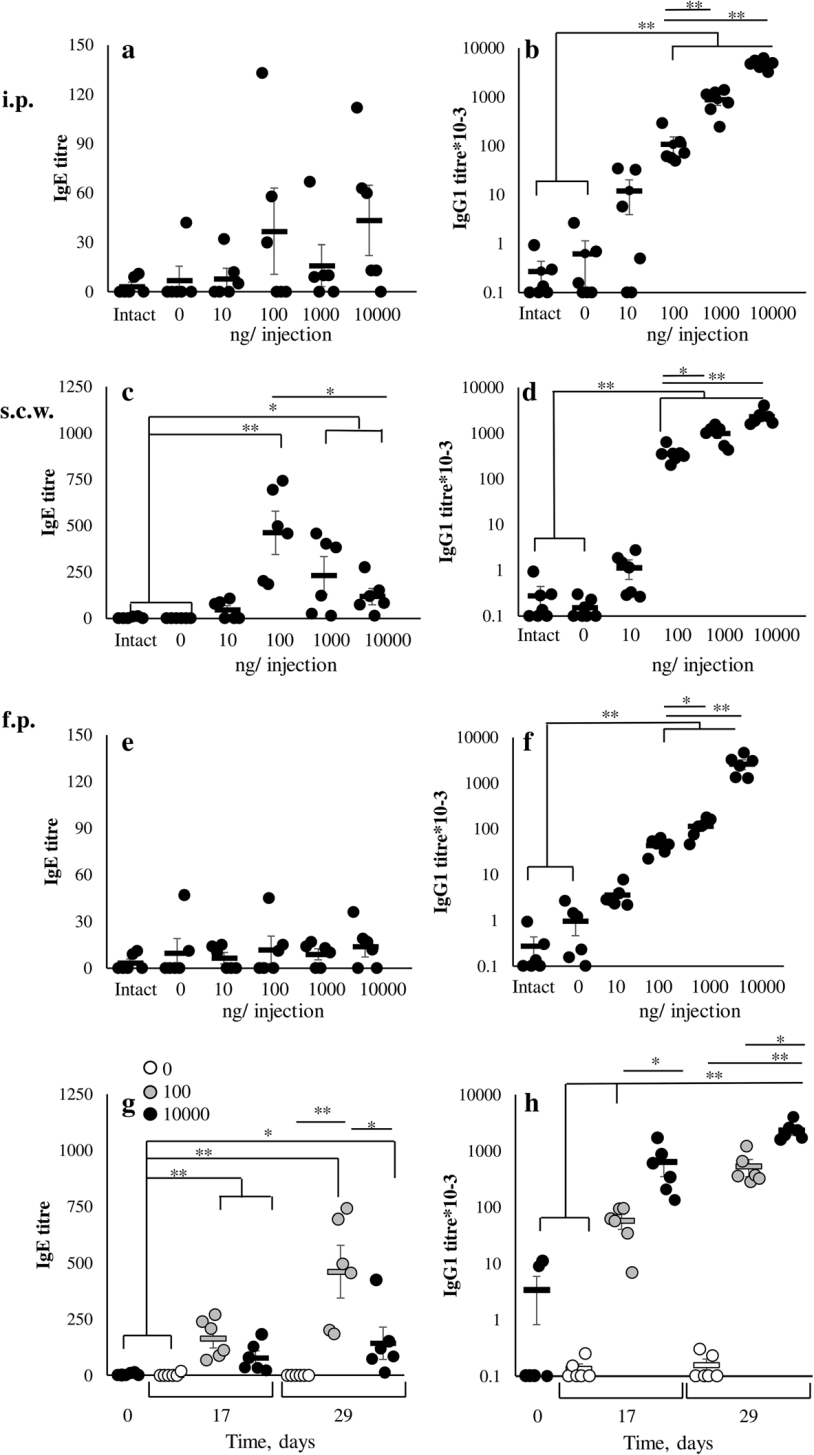
Specific IgE, but not IgG_1_ production, is induced mostly by low antigen doses, and depends on the immunization site in BALB/c mice. BALB/c mice (*n* = 45) were immunized by OVA in different doses (10, 100, 1000, and 10,000 ng/injection) intraperitoneally (i.p.), subcutaneously in withers region (s.c.w) or in the foot pad (f.p.). Antigen administration was performed three times a week for 5 weeks. **a**-**f**: OVA specific IgE (**a**,**c**,**e**) and IgG_1_ (**b**, **d**, **f**) production after 14th immunizations compared to preimmune (intact) or saline immunized mice. **g**-**h**: Time-dependent OVA specific IgE (**g**) and IgG1 (**h**) response in saline (white circles), 100 ng (grey circles) or 10,000 ng (black circles) immunized mice after 7th or 14th immunizations. Data represent mean values ±SD from three independent experiments, (*n* = 5 mice per group). Mann-Whitney test was used for *p* value estimation.*p < 0.05; **p < 0.01 from preimmune and saline immunized mice

The results (Fig. 1 a) show that specific IgE production was induced only after chronic antigen administration in the withers region and mostly in 100 ng low dose group. Some mice after i.p. or f.p. immunization have detectable levels of IgE antibody titres while these effects were statistically insignificant. Specific IgE production was already significant in s.c.w. group after 7th immunization, and at this time point was comparable both in high (10 μg) and low (100 ng) dose groups. However, after 14th immunization it was markedly enhanced in low dose group and only slightly in high dose group (Fig. 1, b).

**Fig. 2:**
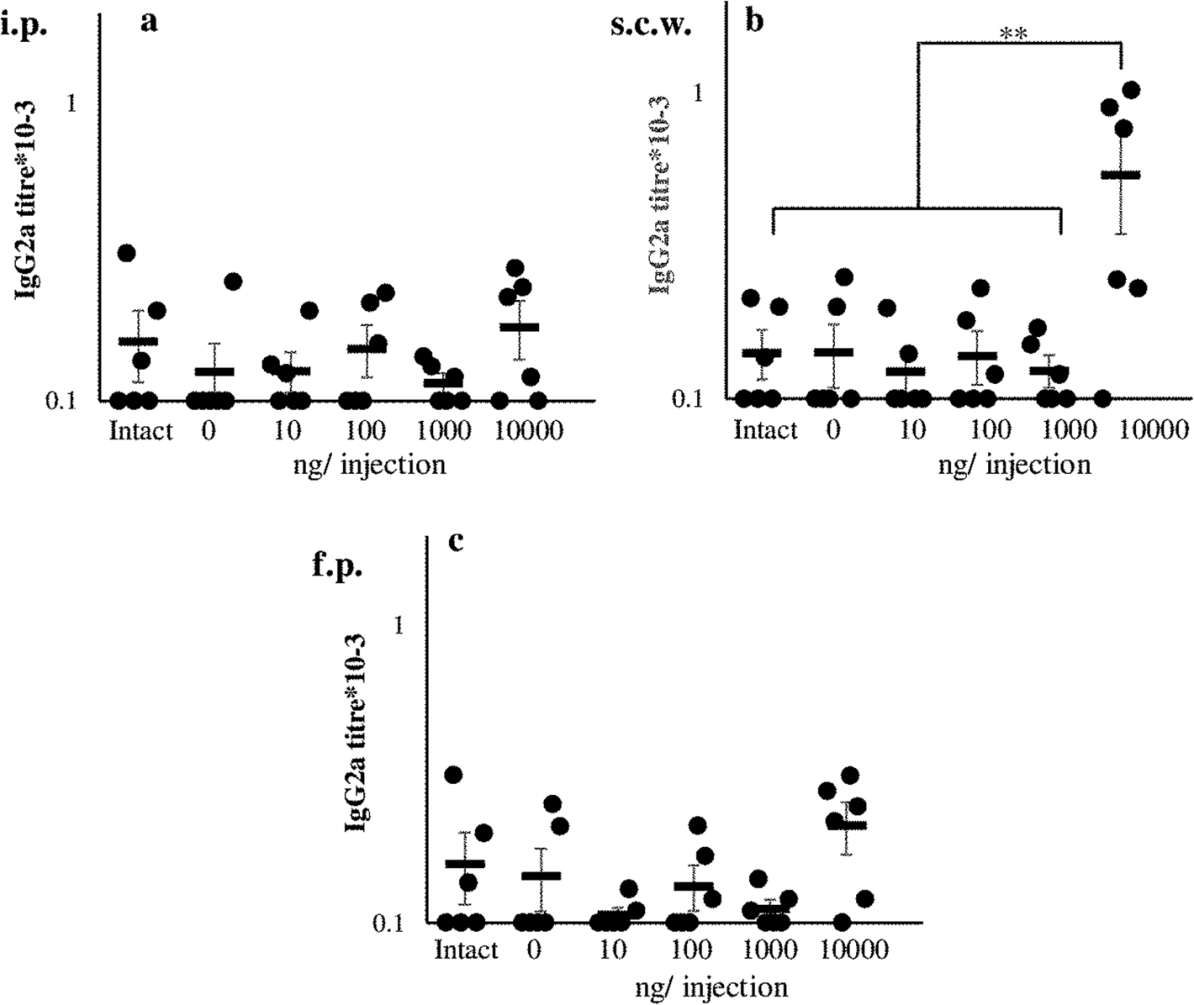
Specific IgG2a production is not induced during long-term low dose immunization regardless administration route. IgG2a production after immunization i.p (**a**), s.c.w. (**b**), or f.p. (**c**) with different doses of OVA (see the details in the legend to Fig. 1)

Specific IgG1 production (Fig. 1, a) was found in all immunized groups while the titers were significantly higher in the high dose groups. IgG1 production was comparable between the high dose groups immunized into different regions, which probably indicates the same efficacy of antigen delivery to SLOs from all these regions. Chronically administrated low doses of the antigen induced much lower IgG1 titers in comparison with the high dose groups (Fig. 1). The most important observation was that the low and high dose groups differed significantly in the balance between IgE and IgG1 production. Specific IgG2a production was minimal in all groups, and increased significantly after prolonged (14th) immunization with high antigen doses in the withers region (Fig. 2).

### The degree of mice sensitization by low antigen doses is associated mostly with specific IgE production

**Fig. 3:**
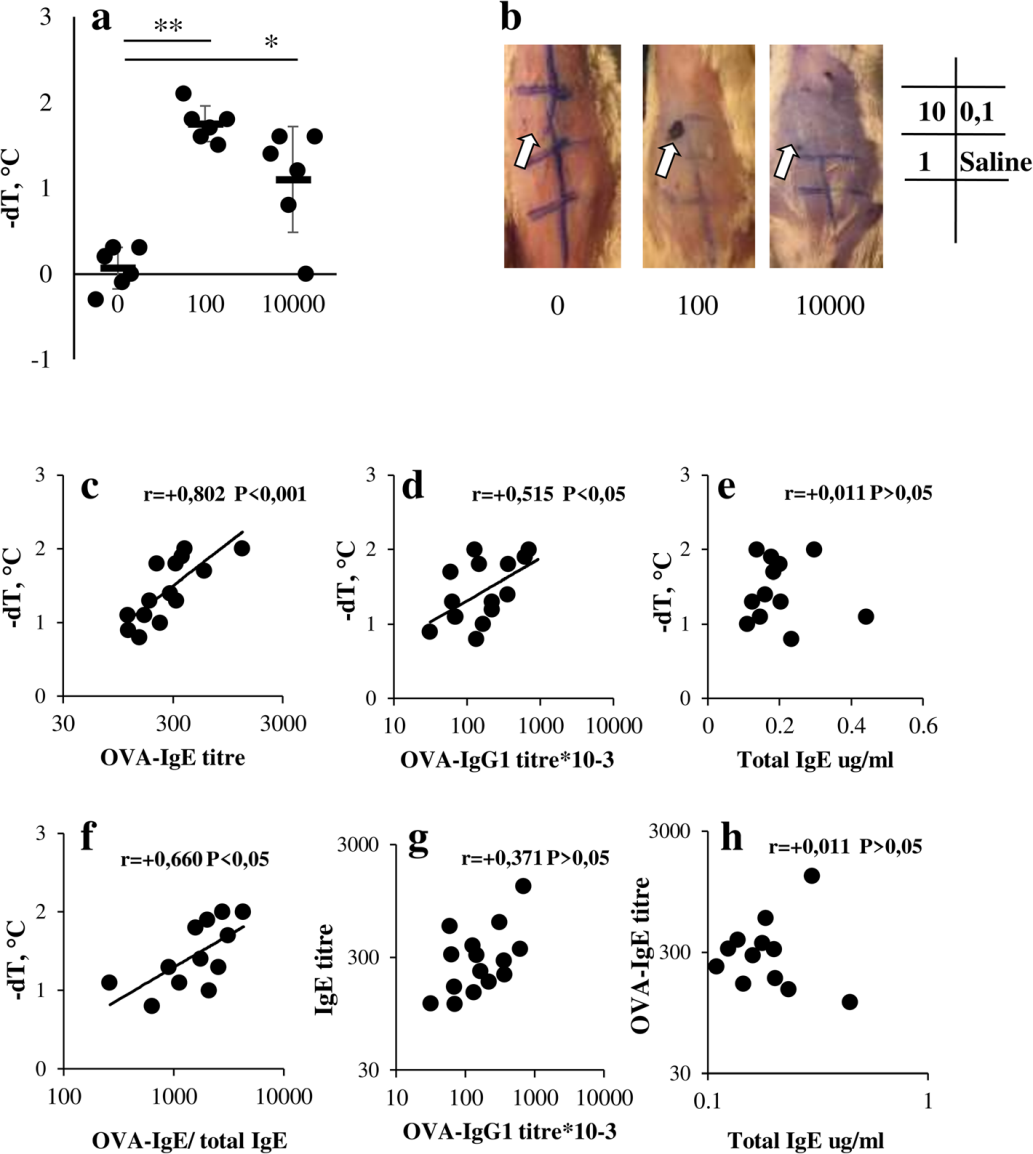
Systematic and local anaphylactic response in BALB/c mice is associated mostly with specific IgE production. **a**: BALB/c mice (*n* = 6–7 per group) intact (0) or immunized with 100 or 10,000 ng/injection OVA in the withers 3 times a week for 4 weeks. A decrease in the temperature (−dT C^o^) was estimated after systematic allergen challenge 3 days after the last OVA immunization by injecting 200 ul of 0,25% OVA i.p. Significance *p < 0.05; **p < 0.01 between indicated groups. **b**: Representative images of Evans Blue local anaphylaxis assay in different immunization groups. Doses in μg of OVA injected are shown. Arrows show the sites of reactions. **c**-**e**: Correlation between specific IgE (**c**), IgG1 (**d**), total IgE (**e**) production and temperature decrease after systematic allergen challenge in low dose (100 ng) immunized mice (*n* = 14). f: Correlations between OVA-specific to total IgE ratio and temperature decrease. **g**: Correlations between specific IgG1 and specific IgE production. **h**: Correlations between specific and total IgE

It is well appreciated that in humans type I hypersensitivity reactions mediated by mast cell and basophil mediators are linked exclusively with allergen-specific IgE antibodies. However, in mice IgG1- and even IgG2a- mediated type I hypersensitivity becomes possible because of basophils degranulation in response to IgG immune complexes [38]. In addition, PAF (Platelet activating factor)-mediated macrophage-dependent anaphylactic response, which closely resembles histamine and leukotriene mediated reactions, becomes possible as well [39]. As mentioned above, in most allergic models currently used, IgE production is induced together with high levels of specific IgG1 production [12–17] which makes clinical situation in these models different from that in human individuals. We presume that our low-dose allergic model differ substantially, namely IgE response is formed without high IgG production. To clarify this, we first estimated the systematic anaphylaxis by measuring skin temperature after the systemic challenge (Fig. 3, a) and local one using Evans blue dye (Fig. 3, b). Mice in low dose groups had more pronounced symptoms of local and systemic anaphylaxis (Fig. 2, a-b).

In the second step we estimated whether systematic anaphylaxis (−dT C^o^) was associated with the specific IgE or IgG1 production. Results (Fig. 3, c-d) demonstrated that the degree of sensitization by systematic challenge directly correlates with both IgE and IgG1 production, but more associated with the elevated OVA-specific IgE levels. During the course of allergic sensitization not only specific but also total IgE levels rose significantly. However the impact of IgE antibodies with unknown specificity on antigen-induced mast cell activation may be negative especially in the case when specific IgE production is low [40]. We have shown that the systematic anaphylaxis did not correlate with the total IgE levels (Fig. 3, e) but there was a correlation with the ratio between specific and total IgE levels (Fig. 3, f). Of note that despite the common mechanisms of induction, specific IgE is produced independently of IgG1 (Fig. 3, g) and the same is observed in the case of total and specific IgE (Fig. 3, h).

### B-cell activation by low antigen doses occurs exclusively in the site of antigen administration

**Fig. 4:**
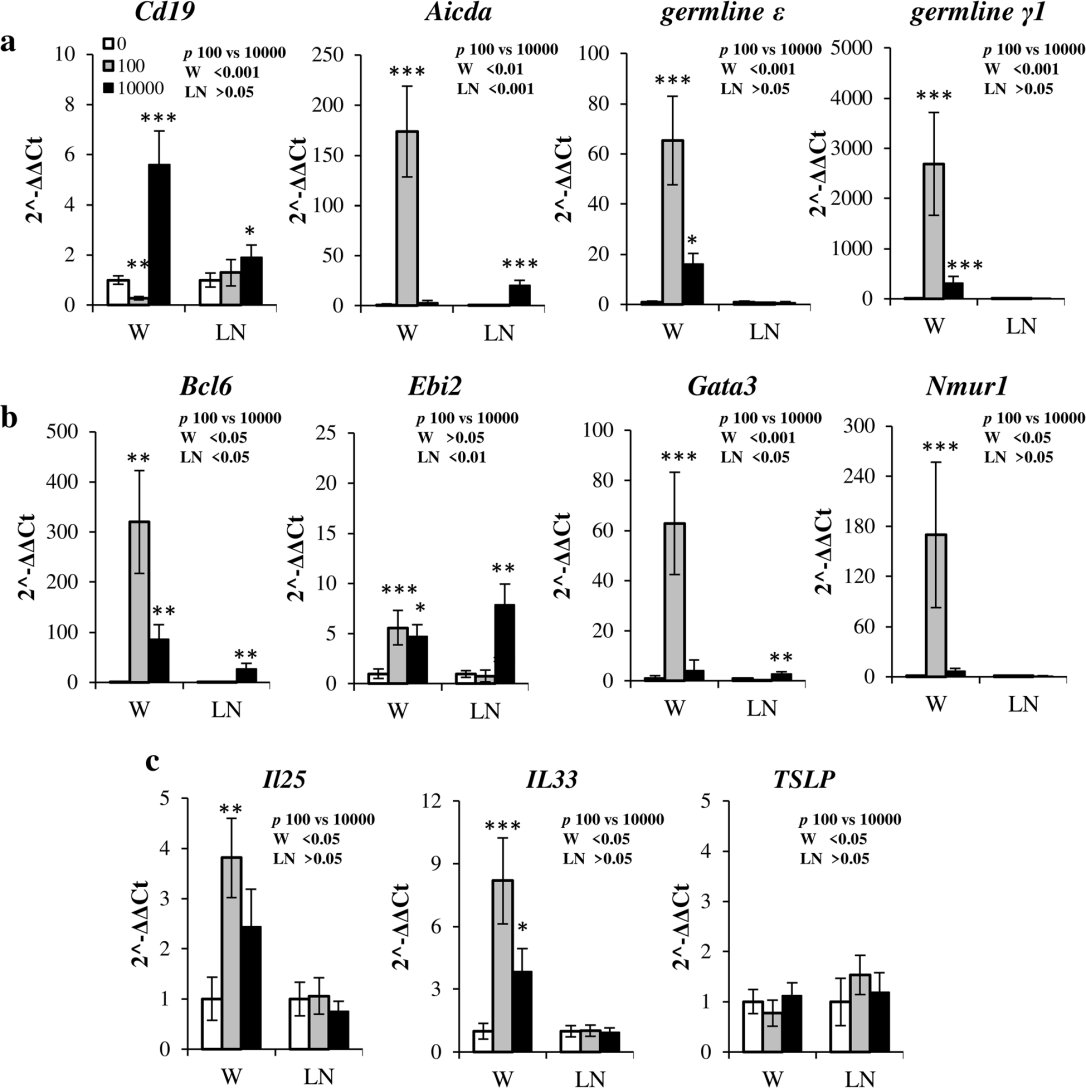
B-cell activation in low-dose immunization protocol occurs in the withers adipose tissue, but not in the lymph nodes. **a**: Expression of B-cell specific gene *cd19* and the genes associated with B-cell antibody class switching *Aicda*, *germline ε* and *γ1* in the withers (W) or axillary lymph nodes (LN) after 14th s.c.w. with saline (0), 100 or 10,000 ng/injection of OVA. All data were normalized to intact control. **b**: expression of genes linked with immune cell activation *bcl6, ebi2, gata3,* and *nmur1*. **c**: expression of innate-immunity linked pro-allergic cytokines genes *IL-25, 33,* and *TSLP* in the same samples. Data are representative from 3 independent experiments (*n* = 5 mice per group, total number of mice 15). Error bars show SD. Statistical significance between immunized and intact mouse groups is shown with asterisks: * - *p* < 0.05; ** - *p* < 0.01; *** - *p* < 0.001; statistical significance between low and high dose groups is shown in each figure in upper right pannel

To confirm our hypothesis about predominant local B-cell activation in the site of antigen administration, we estimated the expression of genes corresponding to B-cell activation in germinal centers (*Bcl6*) [41] or extrafollicular foci (*Ebi2*) [41]. We have also estimated class switch DNA recombination response in general (*Aicda*) [42] and the switching to the IgE and IgG1 (*germline ε* and *germline γ1* transcripts respectively) [42]. As shown in Fig. 4, a B-cell immunoglobulin class switching induced by low antigen doses occurred exclusively in the withers tissue. In the low dose groups expression of *Aicda*, *germline ε* and *germline γ1* genes were triggered exclusively in the withers tissue but not in the regional lymph nodes. It is interesting that even high antigen doses have not triggered germline transcripts expression in the lymph nodes while they have triggered *Aicda* expression (Fig. 4, a).

It should be mentioned that high but not low antigen doses possibly induce relative B-cell accumulation in the withers tissue while low antigen doses induce B-cell depletion from the withers tissue as can be concluded from a relative drop of *cd19* expression. Both doses of antigen induced comparable levels of extrafollicular B-cell activation marker (*Ebi2*) but low doses induced more pronounced germinal center formation in the withers characterized by *Bcl6* upregulation. However, it is not surprising that high antigen doses induce significant accumulation of both types of activated B-cells in lymph nodes. Predominant expression of transcripts corresponding to B-cell activation in low dose group was observed not only when expression was normalized to *Gapdh* but also when it was normalized to *Cd19 (data not shown)*. B-cell IgE class switching depends on IL-4 and less on IL-13 which could be produced by either T helper 2 or ILC2 cells. Both cell types express transcription factor GATA3 [6, 43], but among all immune cells only ILC2 express NMUR1 [44]. We have measured expression of these genes in the withers and the lymph nodes. Low antigen doses induced substantial increase in *Gata3* and *Nmur1* genes expression in the withers tissue. Only high antigen doses triggered expression of *Gata3* in the lymph nodes (Fig. 4, b). Long time antigen administration via needle induced damage of adipose tissue and, therefore, stimulated the expression of tissue cytokines. Expression of *Il25* (only in low dose group) and *Il33* (both groups) but not *TSLP* was induced (Fig. 4, c). It should be noted that due to normalization to respective control samples taken from the same organ the intensity of expression between the withers and the lymph nodes could not be directly compared. The comparison was performed only between the samples from the same tissue.

### Specific IgE production predominantly occurs in regional lymph nodes after migration of activated IgE-switched B-cells from TLSs

In order to develop new allergen-specific immunotherapy methods based on elimination of IgE-producing B-cells and their precursors, one should know not only the site where IgE antibody isotype switching occurs but also the site of IgE antibody production. Because B-cells from TLSs can recirculate between these structures and SLOs [45, 46] we can not exclude the possibility that the final differentiation of IgE^+^ B-cells into antibody producing cells takes place not only in TLSs but also in SLOs.

**Fig. 5:**
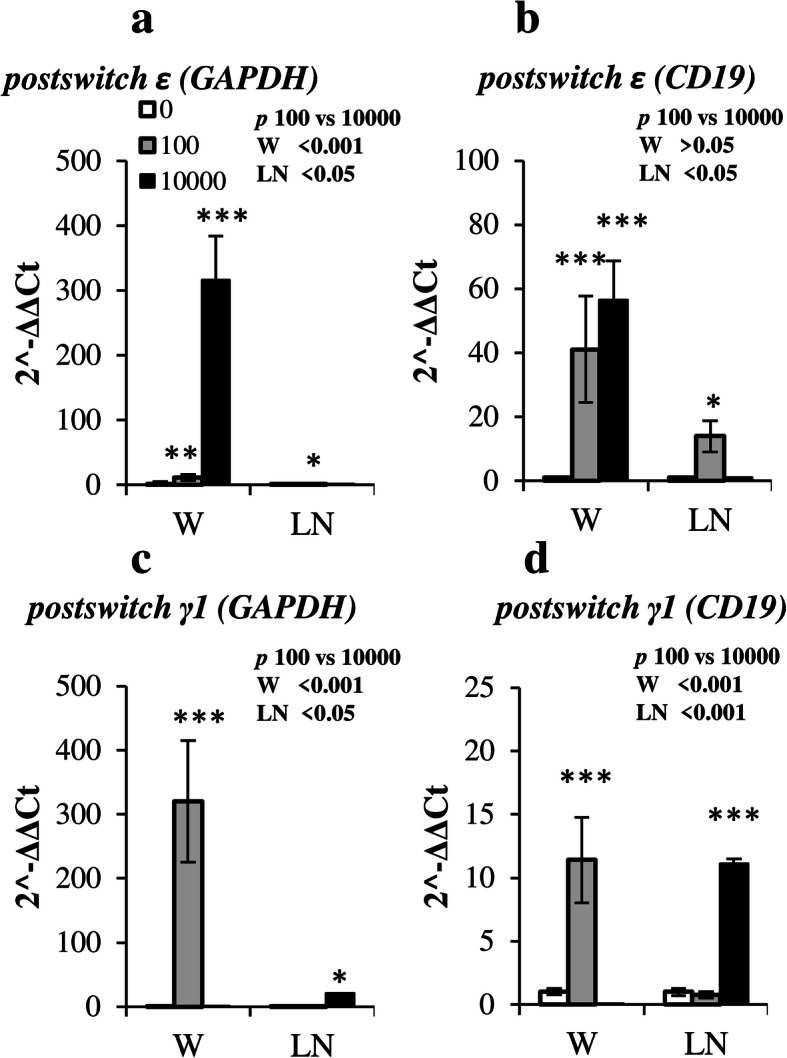
Upon allergen challenge, a portion of antigen-activated B-cells migrates to SLOs where specific IgE production primarily occurs. Expression of postswitch ε (**a**-**b**) or postswitch γ1 (**c**-**d**) transcripts normalized to GAPDH (**a**, **c**) or CD19 (**b**, **d**) in the withers (W) and regional lymph nodes (LN) of intact (0), low-dose (100) and high dose (10000) immunized mice. Data are representative from 3 independent experiments (*n* = 5 mice per group, total number of mice 15). Error bars show SD. Statistics significance between immunized and intact mouse groups: * - *p* < 0.05; ** - *p* < 0.01; *** - *p* < 0.001; statistical significance between low and high dose groups is shown in each figure in upper right pannel

It is well known that due to the presence of low affinity CD23 IgE receptor on B-cells it is relatively difficult to find out truly IgE expressing cells by flow cytometry method [25, 26]. We used *postswitch ε* and *γ1* transcripts as an indicator to find out the location of IgE and IgG1 expressing cells. In contrast to non-coding *germline* transcripts which include specific I_H_ (intervening) promoter following (after their splicing) by C_H_ sequence antibody, coding *postswitch* transcripts expressed after Ig isotype switching starts at their 5ʼ end with a specific pre-rearranged VDJ sequences followed by common intronic non-coding sequences corresponding to iEμ (intronic Enhancer μ) and Iμ chromosomal regions and specific C_H_ coding exons [47]. The primers for these transcripts were designed so that forward primer corresponded to iEμ and Iμ common regions and reverse one to first C_H_ coding exon. Indeed, data from Fig. 5 indicate that the expression of *postswitch ε* and *postswitch γ1* transcripts was induced in the regional lymph nodes. It is not surprising that in a case of regional lymph nodes *postswitch ε* and *postswitch γ1* transcripts were mainly induced by low and high antigen doses, respectively, regardless of the reference gene, i.e. *Gapdh* or *Cd19* (Fig. 5, b and 5, d). However, in adipose tissue the effects of low vs high doses on genes expression were different. When normalized to *Gapdh,* the predominant expression of *postswitch ε,* rather than *postswitch γ1,* in high, rather than low, dose groups was evident (Fig. 5, a, c). When normalized to *Cd19,* the expression of *postswitch ε* transcripts was comparable in both groups (Fig. 5, b, d). Despite inefficient IgE class switch in adipose tissue observed in high dose group, and considering absolute number of B cells rather than percentage of total B cell population, the accumulation of IgE expressing B cells in tissue was more prominent in high vs. low dose group. Together with the data that indicates B-cell depletion from adipose tissue in low but not high dose group (Fig. 4, a), our results suggest that activated B-cells migrate from TLSs to SLOs.

### Dampening of cell proliferation by anti-proliferative drugs enhances specific IgE but not IgG1 production

**Fig. 6:**
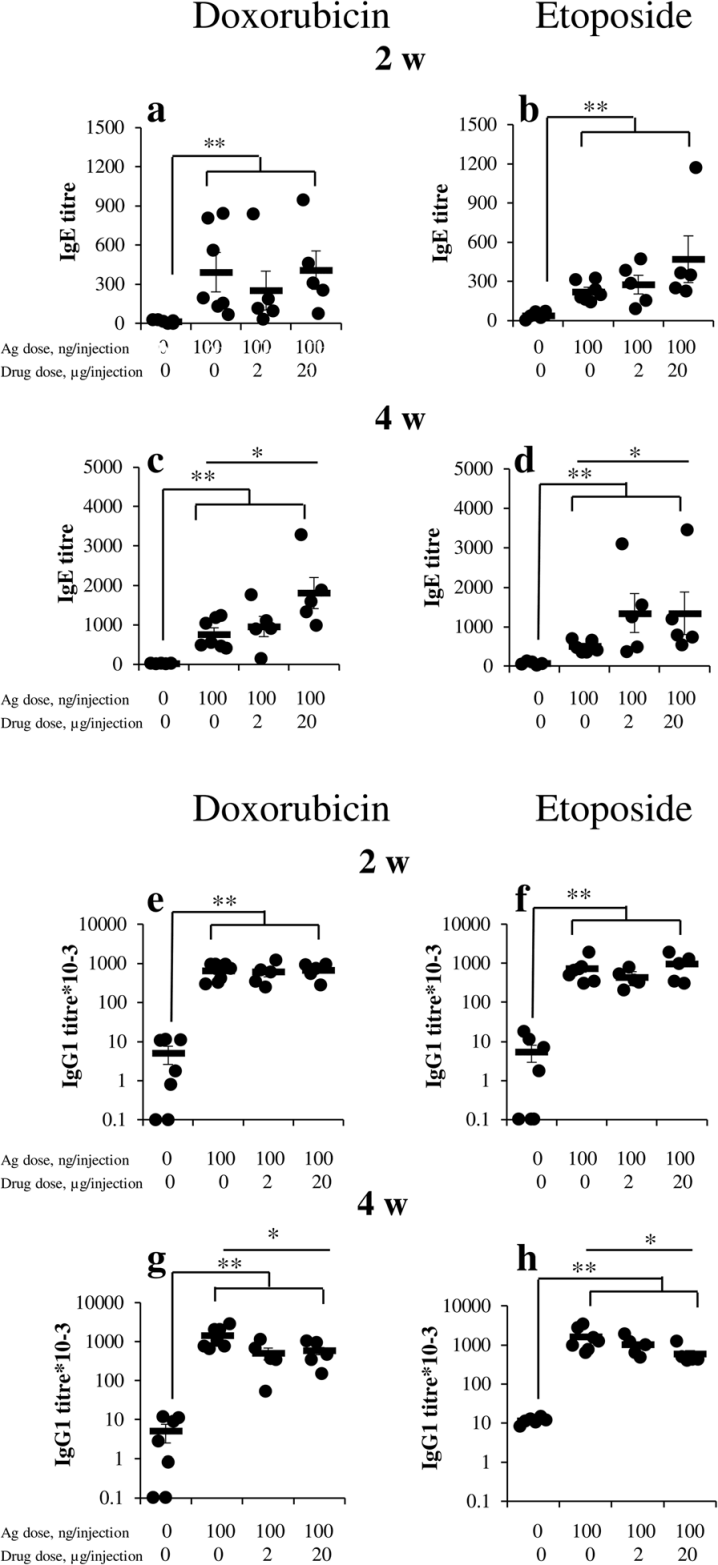
Water soluble and lipophilic anti-proliferative drugs stabilize and enhance specific IgE rather than IgG_1_ production. BALB/c mice were immunized by low (100 ng) dose of OVA alone or with low doses (2 and 20 μg) of anti-proliferative drugs Doxorubicin (D 2 and D 20) and Etoposide (E 2 and E 20) in withers tissue. Three days after the 7th immunization (2 weeks after the start, 2 w) and 3 days after the last immunization (4 weeks after the start, 4 w) blood was taken for the measurement of antigen-specific humoral response. **a**: Specific IgE antibody production. **b**: Specific IgG1 antibody production. Antibody production was compared between saline and immune mice as well as between control (antigen alone immunized) mice and mice immunized together with anti-proliferative drugs. Total number of mice 23, 6 mice in saline and 7 in antigen control group and 5 mice in each test group. Mann-Whitney test was used for *p* value estimation.* - *p* < 0.05; ** - *p* < 0.01 from p.i. and saline immunized mice, § - *p* < 0.05 between bar indicated groups

One of the most interesting observations from data described above is that the expression of *cd19* marker in adipose tissue is downregulated in low dose group compared to control and high dose group, despite the fact that B-cell activation in the withers occurs mostly in the low dose group. In contrast, relative CD19 accumulation in the withers of high dose group was accompanied by a relatively weak intensity of B-cell activation and IgE class switching. Because of the harmless nature of OVA diluted in saline the administration of low antigen dose is unlikely to induce necrotic or apoptotic death of B-cells. It is known that activated B-cells tend to migrate between TLSs and SLOs and the data shown in Fig. 4 support this point of view [48]. Upon antigen-based activation, B-cells tend to proliferate as well but the number of B-cells’ niches in TLSs must be more limited than in SLOs. So, it is reasonable to suppose that a restriction in B-cells proliferation caused by a limited number of niches (or limited proliferation supporting factors) in TLSs in contrast to SLOs shifts the balance in the former from IgG1 to IgE production by yet unknown molecular mechanism. To verify this, we used two commercially available anti-proliferative drugs doxorubicin and etoposide. The water-soluble doxorubicin can be rapidly distributed in internal milieu. Etoposide is more hydrophobic and its specific action would be probably more restricted to adipose tissue. Drugs were administrated with OVA. Data from Fig. 6, a indicate that both drugs induced significant (*p* < 0.05) increase in OVA specific IgE production after prolonged (14th immunizations) treatment, though only high drug dose (20 μg/injection) demonstrated a significant effect. After short time treatment (7th immunizations) only etoposide effect was significant. In contrast, specific IgG1 production at short time period was not affected by these drugs but was slightly, though significantly (*p* < 0.05), dampened after long time treatment (Fig. 6, b). Although the drug-induced effects on IgE and IgG1 specific response were not very pronounced in numerical expression (about 2–3 times) they were significant, differed for antibody classes and could not be accidental. This observation supports our hypothesis that dampened B-cell proliferation could shift the balance towards specific IgE rather than IgG1 production.

## Discussion

Previously we have shown that in young human individuals allergic to house mite dust allergen specific IgE production is not associated with the production of other Ig classes [4]. Consequently they can be triggered in the sites other than SLOs germinal centers, for example in TLSs. In the present study, we demonstrated that model antigen OVA at low dose induced specific IgE production only when administrated in TLSs-enriched region, the withers in particular. Fat-associated lymphoid clusters (FALCs) are relatively constant structures [48] in comparison with the inducible bronchial-associated lymphoid tissues (iBALTs) [32, 49] and, so, immunization in FALCs may represent an adequate model of chronic allergen instillation into respiratory tract of patients containing iBALTs. In such patients, iBALTs can be formed due to chronic bacterial or virus infections or even due to inhalation of different particulate matter, though it was shown only in mouse models [32, 49]. As a matter of fact, FALCs are not the same structures as iBALTs and supporting tissues are completely different. While not denying it, we assume that the basic functional principles for these two types of TLSs are generally the same. Due to the presence of ILC2s in FALCs as well as T helper 2 and M2 macrophages [48] the basic mechanisms of IgE production triggering could be similar both in FALCs and iBALTs. It should be emphasized that the main innate immunity linked pro-allergic cytokines such as IL-33 and TSLP are expressed not only in epithelial barriers but in adipose tissue as well [34, 35].

As it was shown low dose of chronically administrated antigen induced more pronounced IgE response not linked with specific IgG1 or IgG2a response. Our allergic model is considered as clinically relevant representative reconstruction of allergy progression in humans. It is also interesting that specific IgE and IgG1 levels in this model were relatively weakly interconnected if any relation even occurred. Despite this fact, low antigen doses trigger IgE and IgG1 class switching in the same anatomical location – white adipose tissue. Withers adipose tissue rather than regional lymph nodes was the only site of B-cell activation upon low dose antigen administration. It was probably due to the lack of antigen delivering to SLOs in case of low doses. In the absence of pattern recognition receptor (PRR) stimuli, dendritic cells are either not activated or activated in a tolerogenic but not immunogenic way, and their maturation and following migration from tissues to SLOs are rare [50]. Therefore, low antigen doses accumulate only in tissues but not in SLOs.

Low antigen doses are very weak immunological stimulus which does not engage PRRs and very weak triggers of FALCs immune cell activation. So, cells activated by low antigen doses in these structures would be stimulated occasionally in the presence of very low amounts of proliferation supporting stimulus (like IL-2, IL-4, TNF-α etc.) and cell attracting chemokines. The latter may intensify activation-induced B cell migration from tissues to SLOs. This hypothesis was confirmed by reduced expression of *cd19* gene despite induction of genes corresponding to B-cell activation and Ig class switching. Because *cd19* gene expression should correspond to the B-cell numbers [51, 52] and the decrease in this number could not be due to toxicity of administrated antigen or due to the cell differentiation into plasma cells, the only remaining explanation is migration of B-cells from withers tissue. Besides Ig class switching could not occur without germline transcripts induction [47] and so lymph node cells that express postswitch transcripts should arise in the place where germline transcripts expression was detected. The withers adipose tissue seems to be the source of such cells in our allergy model.

Similarly, the data on *postswitch ε* and *γ1* transcripts expression indicates that despite the local tissue-restricted B-cell activation IgE production occurs mostly in SLOs, i.e. regional lymph nodes. It is low rather than high antigen doses induced high levels of specific IgE production, according to ELISA, which enhanced the expression of *postswitch ε* transcripts in the lymph nodes. Also the expression of *postswitch ε* transcripts in the lymph nodes normalized to *Gapdh* was relatively weak which probably corresponded to larger numbers of mature antibody producing cells. This reflects the presence and availability of a higher number of B-cell niches in the lymph nodes than in the withers. In mice, upon high dose antigen administration, the migration of IgE-switched B-cells from TLSs to SLOs does not occur or occurs relatively weakly, therefore, the IgE switched B-cells, that not completely differentiate to plasma cells, accumulate in TLSs. These cells are probably responsible for low level IgE production observed in high dose group.

Supposing that (i) B-cell proliferation in TLSs, namely FALCs, especially in response to weak immunological stimulus, is hampered due to low numbers of B-cell supporting niches and (ii) it is FALCs B-cells that are switched to IgE production in a response to low antigen doses, it is logical to speculate that hampered proliferation in FALCs is responsible for shifting the balance towards IgE production. Indeed, in this work, we showed that anti-proliferative drugs, especially hydrophobic (and apparently locally acting) etoposide, enhanced specific IgE production. But the effects of the same drugs on IgG1 production were opposite. These data are in agreement with the recent observations. For example, in reporter transgenic mice expressing IgE fused with fluorescent protein, the isotype switching occurs mostly in early stages of anti-helminthic immune response when B-cells proliferation in germinal centers is not so rapid as at the later stages [25, 26]. IgE^+^ B-cells have diminished expression of anti-apoptotic proliferation supporting transcription factor Bcl6 [25]. In the other hand, recently switched IgE^+^ B-cells have low levels of costimulatory molecules expression and were more apoptosis prone than IgG1^+^ B-cells [26]. It should be noted that these drugs might have other effects on living cells, for example, on the induction of double stranded breaks that are powerful inducers of class switch recombination [53]. However in this case, it would be logical to expect the induction of both IgE and IgG1 class switching. In addition, these drugs may induce genotoxic stress signaling pathways activation and pro-inflammatory cytokine production [54]. The same genotoxic stress double strand breaks activated pathways also responsible for the inhibition of cell proliferation. Although this issue has remained unexplored in this work it can be assumed that activation of signaling pathways associated with genotoxic stress per se may also be responsible for the shifting the balance towards IgE production. It is interesting that in some groups throughout all the experiments, and especially in the experiments with anti-proliferative drugs, we have observed significant data outliers in humoral immune response. We suggest that it may be due to uneven distribution of FALCs in withers adipose tissue.

In conclusion, in our work we introduced a new low-dose reproducible mouse allergy model which can be an alternative to standard allergy models with high antigen dose usage. In this new model all mice produceed significant specific IgE and IgE isotype switching was triggered in the damaged tissue but not in the lymph nodes. So this new model may be more clinically relevant that commonly used high antigen dose and adjuvant based mouse allergy models. We hope that this model may be interesting and useful for further investigations of IgE production triggering. This model may be useful in studying the mechanisms responsible for the more severe course of atopic diseases in overweight individuals in which the excess of adipose tissue may correspond to the excess of numbers of FALCs in which B-cell proliferation relative to SLOs is hampered and IgE switching occurs more effective. Although the authors do not deny that the other mechanisms may also be responsible for IgE+ cells generation in such structures, for example chronic expression of pro-inflammatory cytokines and adipokines, that may be the subject for future investigations.

## Conclusions

In summary, low dose of administrated allergen triggers B-cell activation and IgE class switching exclusively in TLSs in the damaged tissue. High antigen doses weakly activate B-cells in TLSs and weakly, if any induce IgE antibody production. Hampered B-cell proliferation could shift the balance from IgG1 towards IgE switching in these structures. Despite this fact mature IgE producing plasma cells differentiate from immature precursors in SLOs to where they migrate after the activation in TLSs.

## Methods

### Mice

Female wild type BALB/c mice (6–8 weeks old) were obtained from Andreevka Center (Stolbovaya, Russian Federation). Mice were housed in SPF conditions about 2 weeks before experiments. During all experimental procedures mouse were kept in 12-h light dark cycle at room temperature in a special room in vivarium. Mice were housed in plastic cages (10–12 mice for 1 cage) with filings as a bedding material. Mice were fed ad libitum by granular feed. Mice were treated and their health status was monitored according to an approved by IBCh RAS IACUC protocol.

### Immunization and blood sampling

Ovalbumin (OVA, Sigma Aldrich, Darmstadt, Germany) was used as a model antigen. Mice received OVA by intraperitoneal (i.p.) and subcutaneous injections in foot pad (f.p.) or in the withers areas (W). OVA was administrated repeatedly 3 times a week for 4–5 weeks (total 14 immunizations) in low (100 ng/injection) or high (10 μg/injection) doses in total volume of 100 μl saline. Before study the randomization procedure was performed. Two control groups were commonly used in the study, namely intact mice and saline immunized mice (*n* = 5) We used 2 experimental groups for each immunization protocol when studied the dependence of antibody production from the site of immunization and antigen dose (*n* = 5). The number of animals in groups was determined so that for U-test quantification *p* < 0,01 values could be obtained. Blood sampling from retro-orbital sinus was performed after 7th (17th day) or 14th (33th day) of immunization. Blood was collected, coagulated for 15 min at 37 °C and spinned (600 g, 20 min). Serum was collected and stored at − 20 °C prior to use.

In some experiments, mice (*n* = 5) were immunized with low OVA dose with anti-proliferative drugs doxorubicin and etoposide (Sigma Aldrich) in doses of 2 and 20 μg/ml which corresponded to doses 0.1 and 1 mg/kg. These doses are too low in comparison with usually used for treatment of malignancies [55, 56] and very unlikely cause systematic harmful effects. Doxorubicin was administrated in saline and etoposide in the vehicle containing 0,25% DMSO+ 10% PEG-400 because of water insolubility. Two control groups were used, i.e. saline immunized mice and mice immunized with antigen alone. Two experimental groups for each drug (with different drug dose) were used (*n* = 6–7).

### Elisa

Microtiter plates (MaxiSorb®, Costar, USA) were coated with 50 μl of 5 μg/ml OVA solution in PBS (pH = 7.2) and incubated overnight at + 4 °C. Following extensive washing with PBS containing 0.05% Tween-20 (PBS-T) and subsequent blocking with 5% BSA in PBS, plates were incubated with serially diluted serum samples at + 4 °C overnight. Next day plates were subsequently exposed to primary biotin-labeled anti-mouse antibodies (BioLegend, San Diego, CA, USA) to either IgEa (clone UH297) or IgG1 (clone RMG1 1) or IgG2a (clone RMG2a 62), and streptavidin-conjugated HRP. Plates were further processed with substrate solution (3,3^′^,5,5^′^-tetramethylbenzidine) Optical densities (OD) were measured with automatic 96-well plate reader (Thermo Fisher Scientific, Waltham, MA, USA) at 450 nm with subtraction of optical density at 620 nm that does not correspond to the reaction colored product. Antibody quantities were estimated as OVA-specific antibody titers defined as the serum dilution, in accordance according to 4-PL regression analysis.

For estimation of total IgE levels, plates were coated with unlabeled anti-mouse IgE (clone RME1, BioLegend). Serum samples were added in 1:100 dilution in duplicates with different dilutions of mouse IgE κ (MEA-36, BioLegend). In following steps biotin labeled anti-mouse IgE (UH297) and streptavidin-HRP were added subsequently as described above.

### Systematic anaphylactic response measurement

For measurement of systematic anaphylactic response in mice after full immunization protocol, OVA in challenging dose (0.2 ml of 2.5 mg/ml solution) was administrated i.p. to animals. Body temperature was measured with infrared thermometer CEM DT-8806S, (SEM Test Instruments, Moscow, Russia) as it was performed in [57] before OVA administration and 45 min after. The difference in the temperature between two time points was defined. The experiment was performed in laboratory.

### Local anaphylactic response measurement

Standard Evans Blue based test was used to measure local anaphylaxis response. Briefly, abdominal region of each mouse was shaved and after that different doses stating from 10 μl of 1 mg/ml OVA were administrated subcutaneously in this area. After 3 min 200 μl of 0.5% Evans Blue solution were administrated in the tail vein. After 15 min reaction intensity was evaluated.

### Gene expression measurement by qPCR

After 14th immunization mice were sacrificed in laboratory by cervical dislocation. Lymph nodes were harvested, homogenized and cells were spinned in PBS (600 g, 10 min), followed by the RNA extraction using phenol chloroform based extraction protocol. (ExtractRNA, Evrogen, Moscow, Russia). In the case of white adipose tissue small pieces were harvested and washed from blood in the PBS and then subjected to RNA extraction with the same reagent. cDNA was prepared from the samples with RevertAid First Strand cDNA Synthesis Kit (Thermo Fisher Scientific). Quantitative PCR was performed with SybrGreen I based kits from BioLabMix (Novosibirsk, Russia). Expression of target genes was estimated by normalizing to expression of house-keeping gene GAPDH and was calculated as 2^-d(dCt) in comparison with expression in the tissues of intact control mouse. In the case of postswitch ε transcripts for analysis of absolute IgE-producing B cell numbers in samples, normalization to GAPDH was performed. For measurement of IgE-producing B cell content in B cell fraction, normalization to CD19 was performed. Primers were designed and synthesized by EvroGen. The following primers were used:

for GAPDH F: GGTGCTGAGTATGTCGTGGA; R: TGGAAGAGTGGGAGTTGCTG;

IL-25 F: CCCAGCAAAGAGCAAGAACC; R: ATCCTCTAGCAGCACAAGCG;

IL-33 F: GTCTCCTGCCTCCCTGAGTA; R: GTGGTGCCTGCTCTTCTGAA;

TSLP F: CTGCCTGAATCAAACCTCACAA; R: TGACTGCCCGAACTGTCATT;

CD19 F: TTCACTACTGAGCCTAAGCCTTG; R: CAACAGCCAGAGCCACACT;

AICDA F: ACAGCACTGAAGCAGCCTT; R: GCCCAGCGGACATTTTTGAA;

Bcl6 F: CAGTCCCCACAGCATACAGA; R: CCTCAGAGAAACGGCAGTCA;

EBI2 F: CATAAAAGGACGCCTGCTCG; R: TTGCCAGTGGGGTAGTGAAA;

germline ε F: GCACAGGGGGCAGAAGAT; R: CCAGGTCACAGTCACAGGAT;

germline γ1 F: CACGGGAGATTGGGAAGGAG; R: TTTGGGCAGCAGATCCAGG;

Gata3 F: TGGAGGAGGAACGCTAATGG; R: GATGTGGCTCAGGGATGACA;

NMUR1 F: ATGACTCCTCCCTGCCTCA; R: GAGCACCAGCATATCGGACA;

postswitch ε F: CAGGCTGCTGCTGGGTAG; R: GCTGGTGGTGACCTTGAGTT;

postswitch γ1 F: ACATGCTCTGTGTGAACTCCC; R: AGGTCAGACTCCAGGACAGC. Ct values were determined for each sample. Reaction was performed in DT Prime Amplificator (DNA Technology, Moscow, Russia) according to the following protocol: initial denaturation at + 95 °C for 3 min and then 50 cycles with: 15 s denaturation at + 95 °C, 45 s annealing and elongation at + 60 °C.

### Statistics

For comparisons between experimental groups and samples, nonparametric Mann-Whitney test was used. Levels of *p* < 0.05 were considered statistically significant. For correlation coefficients determination, Spearman test was used. The data from each individual animal was considered as a single point. Mean and standard deviations for each compared group were calculated.

## Data Availability

The datasets obtained and analysed during the current study are available from the corresponding author on reasonable request.

## Abbreviations

CFA: Complete Freundʼs Adjuvant
ELISA: Enzyme Linked Immunosorbent Assay
FALCs: Fat associated lymphoid clusters
f.p.: Foot pad
iBALT: Inducible bronchial-associated lymphoid clusters
ILC2: Innate type 2 lymphoid cells
i.p.: Intraperitoneal
LPS: Lipopolysaccharide
OVA: Ovalbumin
s.c.: Subcutaneous
SLOs: Secondary lymphoid organs

## References

[1] Matricardi PM. The allergy epidemic. In: Akdis CA, Agache I, editors. Global Atlas of Allergy. Zurich: EAACI; 2014. p. 112–4.

[2] Han H, Roan F, Ziegler SF (2017). The atopic march: current insights into skin barrier dysfunction and epithelial cell-derived cytokines. Immunol Rev.

[3] Fujita H, Soyka MB, Akdis M, Akdis CA (2012). Mechanisms of allergen-specific immunotherapy. Clin And Transl Allergy.

[4] Svirshchevskaya E, Fattakhova G, Khlgatian S, Chudakov D, Kashirina E, Ryazantsev D (2016). Direct versus sequential immunoglobulin switch in allergy and antiviral responses. Clin Immunol.

[5] Licona-Limon P, Kim LK, Palm NW, Flavell RA (2013). Th2, allergy and group 2 innate lymphoid cells. Nat Immunol.

[6] Mjosberg J, Bernink J, Golebski K, Karrich JJ, Peters CP, Blom B (2012). The transcription factor GATA3 is essential for the function of human type 2 innate lymphoid cells. Immunity..

[7] Peters U, Dixon A, Forno E (2018). Obesity and asthma. J Allergy Clin Immunol.

[8] Everaere L, Yahia SA, Boute M, Audoussette C, Chenivesse C, Tsicopolous A (2017). Innate lymphoid cells at the interface between obesity and asthma. Immunology..

[9] Shore SA, Shwartzman IN, Mellema MS, Flynt L, Imrich A, Johnston RA (2005). Effect of leptin on allergic airway responses in mice. J Allergy Clin Immunol.

[10] Shore SA, Terry RD, Flynt L, Xu A, Hug C (2006). Adiponectin attenuates allergen-induced airway inflammation and hyperresponsiveness in mice. J Allergy Clin Immunol.

[11] Benezech C, Luu N-T, Walker JA, Kruglov AA, Loo Y, Nakamura K (2015). Inflammation-induced formation of fat-associated lymphoid clusters. Nat Immunol.

[12] Hansen JS, Larsen ST, Poulsen SK, Nielsen GD (2007). Adjuvant effects of inhaled mono-2-ethylhexyl phthalate in BALB/cJ mice. Toxicology..

[13] Clausen SK, Berggvist M, Poulsen LK, Poulsen OM, Nielsen GD (2003). Development of sensitisation or tolerance following repeated OVA inhalation in BALB/cJ mice. Dose-dependency and modulation by the Al(OH)3 adjuvant. Toxicology..

[14] Kim YH, Yoshimoto M, Nakayama K, Tanino S, Fujimura Y, Yamada K, Tachibana H (2013). Tannic acid, a higher galloylated pentagalloylglucose, suppresses antigen-specific IgE production by inhibiting ɛ germline transcription induced by STAT6 activation. FEBS Open Biol.

[15] van der Ventel ML, Nieuwenhuizen NE, Kirstein F, Hikuam C, Jeebhay MF, Swoboda J (2011). Differential responses to natural and recombinant allergens in a murine model of fish allergy. Mol Immunol.

[16] Chang Y-S, Kim Y-K, Jeon SG, Kim S-H, Kim S-S, Park HW (2013). Influence of the adjuvants and genetic background on the asthma model using recombinant Der f 2 in mice. Immune Netw.

[17] Samuelsen M, Nygaard UC, Lovik M (2008). Allergy adjuvant effect of particles from wood smoke and road traffic. Toxicology..

[18] Niederberger V, Niggemann B, Kraft D, Spitzauer S, Valenta R (2002). Evolution of IgM, IgE and IgG (1-4) antibody responses in early childhood monitored with recombinant allergen components: implications for class switch mechanisms. Eur J Immunol.

[19] Resch Y, Michel S, Kabesch M, Lupineck C, Valenta R, Vrtala S (2015). Different IgE recognition of mite allergen components in asthmatic and non-asthmatic children. J Allergy Clin Immunol.

[20] Pereira FA, Silva DA, Cunha-Junior JP, Almeida KC, Alves R, Sung SJ, Taketomi EA (2005). IgE, IgG_1_, and IgG_4_ antibody responses to Blomia tropicalis in atopic patients. Allergy..

[21] Miranda DO, Silva DA, Fernandes JF, Queiros MJ, Chiba CF, Ynoue LH (2011). Serum and salivary IgE, IgA, and IgG4 antibodies to Dermatophagoides pteronyssinus and its major allergens, Der p1 and Der p2, in allergic and nonallergic children. Clin Dev Immunol.

[22] Ecki-Dorna J, Villazala-Merino S, Linhart B, Karaulov AV, Zhernov Y, Khaitov M (2019). Allergen-Specific Antibodies Regulate Secondary Allergen-Specific Immune Responses. Front Immunol.

[23] Aalberse RC, Lupineck C, Siroux V, Nadif R, Just J, Bousquet J (2018). sIgE and sIgG to airborne atopic allergens: coupled rather than inversely related responses. Allergy..

[24] Aalberse RC, Platts-Mills TA, Rispens T (2016). The developmental history of IgE and IgG4 antibodies in relation to Atopy, Eosinophilic esophagitis, and the modified Th2 response. Curr Allergy Asthma Rep.

[25] Talay O, Yan D, Brightbill HG, Straney EM, Zhou M, Ladi E (2012). IgE+ memory B cells and plasma cells generated through a germinal-center pathway. Nat Immunol.

[26] Yang Z, Sullivan BM, Allen C (2012). Fluorescent in vivo detection reveals that IgE+ B cells are restrained by an intrinsic cell fate predisposition. Immunity..

[27] Fiset PO, Cameron L, Hamid Q (2005). Local isotype switching to IgE in airway mucosa. J Allergy Clin Immunol.

[28] Gevaert P, Nouri-Aria KT, Wu H, Harper CE, Takhar P, Fear DJ (2013). Local receptor revision and class switching to IgE in chronic rhinosinusitis with nasal polyps. Allergy..

[29] Gona-Hopler M, Pfaller B, Argeny J, Kanolzer S, Gruber S, Schmidthaler S, Renner S (2017). Aspergillus fumigatus-specific immunoglobulin levels in BALF of CF patients. ERJ Open Res.

[30] Yadava K, Bollyky P, Lawson LA (2016). The formation and function of tertiary lymphoid follicles in chronic pulmonary inflammation. J Immunol.

[31] Hwang JY, Rendall TD, Silva-Sanchez A (2016). Inducible bronchus-associated lymphoid tissue: taming inflammation in the lung. Front Immunol.

[32] Foo SY, Zhang V, Lalwani A, Lynch JP, Zhuang A (2015). Lam, et al. regulatory T cells prevent inducible BALT formation by dampening neutrophilic inflammation. J. Immunol..

[33] Schoetti T, Fisher IP, Ussar S (2018). Heterogeneity of adipose tissue in development and metabolic function. J Exp Biol.

[34] Zeida M, Wernly B, Demyanets S, Kaun C, Hammerle M, Schranz M (2013). Severe obesity increases adipose tissue expression of interleukin-33 and its receptor ST2, both predominantly detectable in endothelial cells of human adipose tissue. Int J Obes.

[35] Turcot V, Bouchard L, Faucher G, Garneau V, Tchernof A, Deshaies Y (2012). Thymic stromal lymphopoietin: an immune cytokine gene associated with the metabolic syndrome and blood pressure in severe obesity. Clin Sci (Lond).

[36] Moro K, Yamada T, Tanabe M, Takeuchi T, Ikawa T, Kawamoto K (2010). Innate production of Th2 cytokines by adipose tissue-associated c-Kit1Sca-11 lymphoid cells. Nature..

[37] Molofski AB, Nussbaum JC, Liang H-E, van Dyken SJ, Cheng LE, Mohapatra A (2013). Innate lymphoid type 2 cells sustain visceral adipose tissue eosinophils and alternatively activated macrophages. J Exp Med.

[38] Tsujimura Y, Obata K, Mukai K, Shindou H, Yoshida M, Nishikado H (2008). Basophils play a pivotal role in immunoglobulin-G-mediated but not immunoglobulin-E-mediated systemic anaphylaxis. Immunity..

[39] Beutier H, Gillis CM, Iannascoli B, Godon O, England P, Sibilano R (2017). IgG subclasses determine pathways of anaphylaxis in mice. J Allergy Clin Immunol.

[40] Martin RK, Damle SR, Valentine EA, Zeliner MP, James BN, Lownik JC (2018). B1 cell IgE impedes mast cell-mediated enhancement of parasite expulsion through B2 IgE blockade. Cell Rep.

[41] Enoksson SL, Grasset EK, Hagglot T, Mattsson N, Keiser Y, Gabrielsson S (2011). The inflammatory cytokine IL-18 induces self-reactive innate antibody responses regulated by natural killer T cells. Proc Natl Acad Sci U S A.

[42] Muramatsu M, Kinoshita K, Fagarasan S, Yamada S, Shinkai Y, Honjo T (2000). Class switch recombination and hypermutation require activation-induced cytidine deaminase (AID), a potential RNA editing enzyme. Cell..

[43] Kanhere A, Hertweck A, Bhatia U, Gokmen MR, Perucha E, Jackson I (2012). T-bet and GATA3 orchestrate Th1 and Th2 differentiation through lineage-specific targeting of distal regulatory elements. Nat Commun.

[44] Cardoso V, Chesne J, Ribeiro H, Garsia-Cassani B, Carvalho T, Bouchery T (2017). Neuronal regulation of type 2 innate lymphoid cells via neuromedin U. Nature..

[45] Schwartz-Cornil I, Epardaud M, Albert JP, Bourgeois C, Geratd F, Raoult I, Bonneau M (2005). Probing leukocyte traffic in lymph from oro-nasal mucosae by cervical catheterization in a sheep model. J Immunol Methods.

[46] Rayamajhi M, Delgado C, Condon TV, Riches DW, Lenz LL (2012). Lung B cells promote early pathogen dissemination and hasten death from inhalation anthrax. Mucosal Immunol.

[47] Xu Z, Zan H, Pone EJ, Mai T, Casali P (2012). Immunoglobulin class switch DNA recombination: induction, targeting and beyond. Nat Rev Immunol.

[48] Cruz-Migoni S, Caamano J (2016). Fat-associated lymphoid clusters in inflammation and immunity. Front Immunol.

[49] Kuroda E, Ozasa K, Temizoz B, Ohata K, Koo CX, Kanuma T (2016). Inhaled fine particles induce alveolar macrophage death and interleukin-1a release to promote inducible bronchus-associated lymphoid tissue formation. Immunity..

[50] Dalod M, Chelbi R, Malissen B, Lawrence T (2014). Dendritic cell maturation: functional specialization through signaling specificity and transcriptional programming. EMBO J.

[51] Howe JG, Crouch J, Cooper D, Smith Real-Time BR (2004). Quantitative reverse transcription PCR for Cyclin D1 mRNA in blood, marrow, and tissue specimens for diagnosis of mantle cell lymphoma. Clin. Chem..

[52] Kim D-Y, Lee SH, Carter RG, Kato A, Schleimer RP, Cho SH (2016). A Recently Established Murine Model of Nasal Polyps Demonstrates Activation of B Cells, as Occurs in Human Nasal Polyps. Am J Respir Cell Mol Biol.

[53] Sherman MN, Kuraishi AI, Deshpande C, Hong JS, Cacalano LA, Gatti RA (2010). AID-induced Genotoxic stress promotes B cell differentiation in the germinal center via ATM and LKB1 signaling. Mol Cell.

[54] Demaria M, O’Leary MN, Chang J, Shao L, Alimirah F, Koenig K (2017). Cellular senescence promotes adverse effects of chemotherapy and Cancer relapse. Cancer Discov.

[55] Zocchi E, Tonetti M, Polvani C, Guida L, Benatti U, De Flora A (1989). Encapsulation of doxorubicin in liver-targeted erythrocytes increases the therapeutic index of the drug in a murine metastatic model. Proc Natl Acad Sci U S A.

[56] Lee JS, Takahashi T, Hagiwara A, Yoneyama C, Itoh M, Sasabe T (1995). Safety and efficacy of intraperitoneal injection of etoposide in oil suspension in mice with peritoneal carcinomatosis. Cancer Chemother Pharmacol.

[57] Kawakami Y, Sielski R, Kawakami T. Mouse body temperature measurement using infrared thermometer during passive systemic anaphylaxis and food allergy evaluation. J Vis Exp. 2018;139. 10.3791/58391. 10.3791/58391 PMC6235190 30272668

